# Pre-clinical Models for Malignant Mesothelioma Research: From Chemical-Induced to Patient-Derived Cancer Xenografts

**DOI:** 10.3389/fgene.2018.00232

**Published:** 2018-07-04

**Authors:** Noushin Nabavi, Jingchao Wei, Dong Lin, Colin C. Collins, Peter W. Gout, Yuzhuo Wang

**Affiliations:** ^1^Department of Urologic Sciences, Vancouver Prostate Centre, University of British Columbia, Vancouver, BC, Canada; ^2^Department of Experimental Therapeutics, BC Cancer Research Centre, Vancouver, BC, Canada; ^3^Department of Urology, the Third Xiangya Hospital, Central South University Changsha, China

**Keywords:** rare diseases, genomics, patient-derived xenografts, malignant mesothelioma, pre-clinical cancer research, drug development

## Abstract

Malignant mesothelioma (MM) is a rare disease often associated with environmental exposure to asbestos and other erionite fibers. MM has a long latency period prior to manifestation and a poor prognosis. The survival post-diagnosis is often less than a year. Although use of asbestos has been banned in the United States and many European countries, asbestos is still being used and extracted in many developing countries. Occupational exposure to asbestos, mining, and migration are reasons that we expect to continue to see growing incidence of mesothelioma in the coming decades. Despite improvements in survival achieved with multimodal therapies and cytoreductive surgeries, less morbid, more effective interventions are needed. Thus, identifying prognostic and predictive biomarkers for MM, and developing novel agents for targeted therapy, are key unmet needs in mesothelioma research and treatment. In this review, we discuss the evolution of pre-clinical model systems developed to study MM and emphasize the remarkable capability of patient-derived xenograft (PDX) MM models in expediting the pre-clinical development of novel therapeutic approaches. PDX disease model systems retain major characteristics of original malignancies with high fidelity, including molecular, histopathological and functional heterogeneities, and as such play major roles in translational research, drug development, and precision medicine.

## Introduction

Malignant mesothelioma (MM) as a rare disease occurs infrequently in the general population, typically affecting fewer than 3,000 patients in North America (Bianchi and Bianchi, 2014). The pleural form, affecting the lining of the chest cavity and lungs, is often referred to as a man-made disease due to high correlation of incidence with exposure to asbestos. The rarer form affecting the abdominal cavity, i.e., peritoneal mesothelioma (PeM), is more common in women and is often subject to incorrect diagnosis (Alakus et al., 2015; Shin and Kim, 2016). Additionally, aside from a few large scale-studies on pleural mesothelioma (PM) (Zhang et al., 2015; Bueno et al., 2016; Joseph et al., 2017), PeM remains largely unexplored. Like many known rare diseases, mesotheliomas have no approved targeted therapy and cisplatin-pemetrexed chemotherapy remains the standard of care (van Zandwijk et al., 2013). MM is frequently acute and life-threatening, with survival of less than 1 year in the majority of cases. While large numbers of people may have been exposed to asbestos through occupational or domestic exposure, significantly smaller numbers go on to develop mesothelioma (Carbone et al., 2012), suggesting the involvement of genomic predispositions in disease development. For instance, recent genomic profiling of mesotheliomas shows that mutations in the *BAP1* gene render its protein product inactive, and are correlated with MM and uveal melanoma incidence (Testa et al., 2011; Alakus et al., 2015; Ji et al., 2016). Whereas more research is needed to understand other genetic links to MM tumorigenesis, progress is exacerbated by its existential paradox, lack of funding, disease model systems and research resources. Next-generation sequencing technologies (ChIP-Seq, RNA-Seq, DNA-Seq, and Proteome-Seq) applied to patient-derived cell and animal models in rare disease research are becoming key venues to identify the underlying etiology of the disease. Here we review the past and current pre-clinical models in MM research (see **Supplementary Table S1**) and address some of the challenges, limitations, and opportunities that can advance its status quo.

## Historical Development of Mm Models Through Chemical Induction and Gene Modification

It is well-established that chronic exposure to asbestos induces development of human pleural mesothelial cells with cancer-like properties (Lohcharoenkal et al., 2013). Clinically, it has been demonstrated that exposure to asbestos causes many lung diseases such as asbestosis, MM, and lung cancer due to the generation of chromosomal damage and DNA aberrations (Nymark et al., 2007). Historically, to study tumorigenesis of MM, animal and cell models were induced through exposure to varying doses and sizes of asbestos fibers (Whitaker et al., 1984; Topov and Kolev, 1987; Davis et al., 1992; Pass and Mew, 1996) by intrapleural or intraperitoneal injection of asbestos fibers into laboratory rats, mice, or hamsters or incubation of normal mesothelial cell lines with the fibers. Potential MM models would eventually manifest following long latency periods of approximately 7 months for mice, 12 months for rats, and years for primates (Suzuki, 1991). Although these models are difficult to develop, they are ideal platforms for testing and selecting new combinations or targeted therapies, or studying *de novo* carcinogenic pathways.

Prior to the turn of this century, Simian virus 40 (SV40) was another identified agent widely studied to induce MM (Testa et al., 1998; Bocchetta et al., 2000). Although it is controversial that SV40 contributes to the development of mesothelioma as a causative factor (Hubner and Van Marck, 2002; López-Ríos et al., 2004), its role as a cofactor with asbestos has been established in animal models. Interestingly, some studies showed that SV40 rendered animals more susceptible to asbestos-related carcinogenesis (Kroczynska et al., 2006; Robinson et al., 2006), while asbestos was also reported to promote SV40 infection of cells (Appel et al., 1988).

Following chemical induction of MM, novel genetic models were generated to understand genomic predispositions to this malignancy independent of exposure to asbestos (Jongsma et al., 2008). Both knock-out and knock-in animal models are meaningful steps forward in research and are particularly useful for showing the potential importance of a single gene in disease progression. Well-established genetic studies associated with MM include loss of *p16^INK4A^*, *p14^ARF^*, *Nf2*, *p53* and possibly *Rb* (Cheng et al., 1994; Bianchi et al., 1995; Mor et al., 1997; Papp et al., 2001). Additional studies showed that *Nf2* is one of the most frequently mutated tumor suppressor genes in PeM (Sekido et al., 1995), and that asbestos-exposed *Nf2* knockout mice exhibited accelerated MM tumor formation (Altomare et al., 2005). To demonstrate the powerful effect of *Nf2* deficiency in inducing MM, *Nf2*-deficient mice were crossed with either *Ink4a/Arf*-deficient or *p53*-deficient mice, and in the absence of any exposure to asbestos, a high incidence of short median survival of invasive pleural mesothelioma developed (Altomare et al., 2005; Jongsma et al., 2008). Combined genomics studies further showed that MM tumors have frequent hypermethylations or deletions at the *Cdkn2a/Arf* and *Cdkn2b* gene loci (Kane, 2006). In summary, in combination with patient-derived xenografts, these models are invaluable systems for studying chronic and systemic effects of gene aberration burden in MM development and deciphering clear linkages between asbestos exposure and genetic predisposition.

## Patient-Derived Cell Models of Mm Accelerating Research and Development

Patient-derived cell lines in MM have served as impactful tools for profiling gene expressions, excavating new asbestos-associated genes and pathways, and identifying chromosomal regions that contribute to asbestos and therapy responses. Common chromosomal abnormalities, such as deletions, of chromosomes 1, 3, 4, 9, 11, 14 and 22, have been identified in patient-derived cell lines of MM (Popescu et al., 1988; Taguchi et al., 1993; Lee et al., 1996). Additionally, asbestos-affected genetic pathways such as integrin-mediated signaling pathways, MAPK pathways, and NFKB/IKB pathways (Ramos-Nino et al., 2003) can be attributed to advances brought about by patient-derived cell lines.

These developments started historically as early as 1982 in a study that reported a first-in-field *in vitro* patient-derived mesothelioma cell line that was generated from abdominal fluid of a patient diagnosed with mesothelioma. It was shown that this cell line stably yielded MM up to 100 passages (Behbehani et al., 1982). Subsequently, an H-MESO-1 cell line was derived from a 35-years old male diagnosed with MM (Reale et al., 1987); it was capable of growing both as nodules and as ascitic fluid with peritoneal seeding and diffuse peritoneal thickening, strongly mimicking the growth pattern of this tumor type in humans (Reale et al., 1987). Subsequently, a panel of 17 human MM cell lines was derived from 61 patients (46 effusions, 9 biopsies, and 6 tumors obtained at autopsy) and 5 of these cell lines were characterized to closely recapitulate human disease (Wu et al., 1985; Versnel et al., 1989; Tange et al., 1995). Interestingly, Ishiwata et al. (2003) derived a cell line termed HMMME in 2003 from the pleural fluids of a MM case that grew well, both *in vitro* and *in vivo*, with a doubling time of 42 h, without interruption for 12 years, and was sub-cultured over 200 times. Following these advances, Usami et al. (2006) established and characterized additional malignant PM cell lines (ACC-MESO-1, ACC-MESO-4, Y-MESO-8A, and Y-MESO-8D), and detected differentially expressed genes between Y-MESO-8A and Y-MESO-8D, which were derived from the same patient. Among these four cell lines, *Nf2* was found to be mutated only in ACC-MESO-1. This is an important finding as exploring the genomic aberrations associated of cells is necessary to testing potential targeted therapies and to better translate research discoveries. A search of clinicaltrials.gov in order to find clinical trials treating *NF2* mutated solid tumors in patients suggests Everolimus, an oral derivative of rapamycin (NCT02352844) which is in phase 2 of trials, may be a potential targeted therapy to test in these cells. In another study, homozygous deletions of *p16^INK4A^* and inactivation of the *p14^ARF^* gene were found in all four cell lines. Again, the NCT02688907 phase 2 clinical trial recruiting small cell lung-cancer patients with a *p16^INK4A^* mutation uses AZD1775, a tyrosine kinase inhibitor. *In vitro* studies with this inhibitor in relevant MM cell lines as such can accelerate pre-clinical developments. Additionally, a key advancement in the field was the establishment of three PM cell lines (TCC-MESO-1, TCC-MESO-2, and TCC-MESO-3) by Yanagihara et al. (2010) from primary and metastatic tumors of a patient with epithelioid subtype and 1 line from a mixed tumor subtype (epithelioid and sarcomatoid) allowing for pathological subtype investigations both *in vitro* and *in vivo*. Traditional cell culture technologies such as gene transfections can be widely applied to malignant cells to directly study mechanisms of pathogenesis and tumorigenesis. However, cells in multicellular spheroids can mimic resistance to drugs better than monolayer cells as they preserve the complexity of the original tumor (Yanagihara et al., 2010). Thus, discovering genomic aberrations in these cell lines further enables the assessment and development of pre-clinical targeted therapeutics. One example utilizing testing drugs on patient-derived cell lines is a study confirming the successful response of a 3D multicellular spheroids of MM (MSTO-211H) to cytotoxic Paclitaxel-loaded nanoparticles (Lei et al., 2015). Appreciating the numerous advantages of cell lines in pre-clinical research, they are not without their shortcomings some of which include inability to precisely reflect *in vivo* conditions such as heterogeneities and tumor microenvironment. Thus, they necessitate further validation in models that better mimic intratumoral parameters of human disease.

## Patient-Derived Animal Models of Mm for Pre-Clinical Research and Development

The practice of engrafting tumor fragments from patient surgical tissues or biopsies either heterotopically or orthotopically in immunodeficient mice started in the 1950s (Woolley, 1958). Heterotopical implants occur when the tumor fragments are implanted into mice unrelated to the original tumor site, generally in the subcutaneous site, or sometimes in sub-renal capsular sites. Both of these models are unique in answering specific questions and are invaluable tools for mesothelioma research. Subcutaneous tissue xenografts rarely produce metastasis in mice, and have engraftment success rates of 40–60%, whereas sub-renal capsule tissue xenografts maintain the original tumor stroma (at least in the first generation) as well as the host stroma and have engraftment success rates of 95% (Wang et al., 2017). Knowing this, the mesothelioma field has attempted many of these techniques with remarkable success. For instance, Arnold colleagues for the first time reported inoculation of mesothelioma cells into nude mice to establish an *in vivo* mesothelioma xenograft model in Arnold et al. (1979) and Nissen et al. (1979). Later, Chahinian et al. (1980) successfully established six such xenografts by subcutaneous inoculation of fresh tumor specimens into nude mice. To investigate the suitability of MM PDX models in pre-clinical studies, tumors from 50 patients were implanted into immunodeficient mice and serially passaged for up to five generations (Wu et al., 2017). Successful PDXs were formed in 20 of 50 (40%) tumors implanted retaining both the morphology and characteristic genotypic and phenotypic markers of the primary lesion. Interestingly, PDX formation was associated with poor survival of the patients, making them ideal and replicable models to identify prognostic biomarkers and/or develop better pre-clinical therapeutic strategies. Interestingly, PDX models derived from epithelioid and sarcomatoid pathologies of mesothelioma have similar differentiation states as the original tumors (Darai-Ramqvist et al., 2013). The sarcomatoid mesothelioma subtype present with a faster growth rate than the epithelioid subtype in PDXs, consistent with its aggressive physiological behavior in humans (Darai-Ramqvist et al., 2013). The different growth patterns in mixed type mesotheliomas are suitably replicated in PDXs, making them invaluable models for investigating MM’s cell differentiation, heterogeneity, and tumor evolution. In another study, mesothelioma cells isolated from ascites or pleural fluid of mesothelioma patients were injected into nude/SCID mice to generate PDX models. All PDXs exhibited morphologic and immunohistochemical features consistent with those of original patients’ mesothelioma cells (Kalra et al., 2015). Since these models provide biological incubators for inoculated tumors, they provide a tumor repository platform that allows deep genomic and pathological analyses. For instance, it was found in this study that *BAP1* loss correlated with enhanced tumor growth. Similar to human cells, murine mesothelioma cells injected into humanized BALB/c mice allow study of tumor cell interaction with the immune system. In one study, murine mesothelioma cells responded to exogenous High Mobility Group Box 1 protein, a Damage-Associated Molecular Pattern that acts as a chemoattractant for leukocytes and as a proinflammatory mediator (Mezzapelle et al., 2016). Other malignant mesothelioma cell lines, TCC-MESO-1, TCC-MESO-2 and TCC-MESO-3, show tumorigenicity in mice after orthotopic implantation (Yanagihara et al., 2010) and allow evaluation of anticancer agents *in vivo* (Opitz et al., 2007; Yanagihara et al., 2010). Thus, establishment of PDX models of MM in immunocompromised mice provides a high-fidelity model with minimal genetic drift and physiologically relevant tumor microenvironments to investigate the etiology of this malignancy and develop new therapeutic agents for MM. Here, we hope to shed light on the concept that PDXs in combination with emerging gene-editing or nano-particle therapeutic techniques are paramount to harnessing the full potential of animal models. To our surprise, however, animal studies that take into account the genomic background of MMs for targeted therapy explorations are very limited. We think integrating the knowledge from genomic aberrations of MM models with targeted therapeutics for those aberrations can be largely utilized in mesothelioma research and have the potential for illuminating the value of these models in answering critical and unmet research needs. Some of the unexplored capabilities that these MM PDX models provide include expediting targeted therapy efficacies and accelerating pre-clinical translation of novel therapeutic approaches from other indications and applying them to MM.

## Conclusion

Our understanding of MM biology is hindered by its slow onset, low prevalence, and difficulties of manifesting prolonged predisposing conditions to induce lesions in model systems. While historical models need yet to faithfully recapitulate all aspects of the clinical disease, MM PDX models are remarkable systems that enable insights into the genetics of tumor initiation, growth, and metastasis. In this review, we provide an overview of the major known models in the mesothelioma field that have been instrumental in key discoveries in the past century. We also highlight unresolved questions and limitations that hamper translational progress. We argue that although PDX models come with inherent challenges such as cost, failing to graft *in vitro* or *in vivo*, or not efficiently translating to clinical protocols, they are invaluable platforms to investigate the underlying mechanisms driving tumor initiation, progression, metastatic events, as well as therapeutic interventions. Conventional orthotopic, sub-renal, and subcutaneous transplantation models as well as cell lines remain indispensable in continuing the study of MM and new models that can spontaneously develop mesothelioma and be used to test novel and targeted agents are current clinically unmet needs.

## Author Contributions

NN, JW, DL, PG, CC, and YW: conception. NN, JW, DL, and PG: writing, review, and/or revision of the manuscript. CC and YW: study supervision.

## Conflict of Interest Statement

The authors declare that the research was conducted in the absence of any commercial or financial relationships that could be construed as a potential conflict of interest.

## Supplementary Material

TABLE S1 Summary of pre-clinical models used for malignant mesothelioma research.
